# Advancements of sustainable development goals in co-production for climate change adaptation research

**DOI:** 10.1016/j.crm.2022.100438

**Published:** 2022

**Authors:** Halvor Dannevig, Mari Hanssen Korsbrekke, Grete K. Hovelsrud

**Affiliations:** a Western Norway Research Institute, P.O Box 163, 6851 Sogndal, Norway; b Nordland Research Institute, Universitetsallèen 11, 8049 Bodø, Norway

**Keywords:** Sustainable development goals, Climate change, Adaptation, Co-production, Participatory methods

## Abstract

The United Nations Sustainable Development Goals (SDGs) is a new discursive regime that encompasses global environmental change challenges and sustainability sciences, including adaptation to climate change. Co-production of knowledge has become a key, intrinsic component in both sustainability sciences and adaptation research. In this review article, we investigate if and how the SDG agenda is included in the application of participatory approaches and co-production of knowledge for climate change adaptation. We review findings from such processes in projects whose objective is to foster adaptation in the context of SDGs and to categorize the methods employed to forward co-production. We investigate 1) whether and how co-production approaches localize SDG targets and address tradeoffs and synergies, 2) whether they focus on power asymmetries and political dimensions in such participatory processes, and 3) whether and how the literature show that the SDG agenda contributes to a shift in the role of researchers towards a more interventionist approach to co-production. Our results show that there is little evidence that the SDG agenda contributes to a shift towards more interventionist or transformative approaches within climate change adaptation. Further, we have a identified a missed opportunity in the exclusion of “social” SDGs (SDG 5 and 10) in the discussions of adaptation and co-production and SGDs. Most importantly, we find that participatory efforts, including the co-production of knowledge, for localizing SDG goals and resolving tradeoffs and benefits, are the most salient aspects that tie the three co-production - adaptation - the SDG agenda together. Such participatory localizing processes have a great potential in facilitating long-enduring empowerment and legitimacy in adaptation efforts.

## 1 Introduction

This article reviews the literature that connects the co-production of knowledge for climate change adaptation with the SDGs, with the aim of identifying how and why processes of co-production may foster climate change adaptation in a broader sustainability context. Knowledge co-production is increasingly emerging both as a key for harnessing science for sustainable development (Cash et al., 2003, Clark et al., 2016, Norström et al., 2020) and as a prerequisite for adaptation to climate change (Dilling and Lemos, 2011), referred to here as *adaptation.*

The discrepancy between the stated need for adaptation and the amount of adaptation that takes place has been explained by a dominance of technical fixes and a desire for accurate risk assessments in the adaptation discourse. There has been a tendency to neglect that adaptation is a social process in terms of values, ethics, justice, and different ways of knowing (Nightingale et al., 2019, Castree et al., 2014). Co-production and other participatory approaches have emerged in research on adaptation partially as a response to such criticism, and in recognizing that local and traditional knowledge matters for developing adaptation strategies (e.g., Westskog et al., 2017). At the same time, the scholarly discourses on sustainable development, and increasingly on adaptation, are being shaped by the UN 2030 agenda and the 17 sustainable development goals (SDGs) and the associated targets and indicators. The SDGs are the latest attempt by the UN to shape the global development agenda, following the Local Agenda 21 and then the Millennium Development Goals (MDGs). The SDGs are designed to represent the full range of sustainability issues on a global scale, and every country—rich and poor—is urged to address and achieve them. Climate change-related challenges are addressed in SDG 13, with several targets tied to adaptation. SDG 13.1 calls for “[s]trengthen[ing] resilience and adaptive capacity to climate-related hazards and natural disasters in all countries”; SDG 13.2 calls for the “institutionalization of mitigation and adaptation,” and SDG 13.2 for “improv[ing] education, awareness-raising and human and institutional capacity on climate change mitigation, adaptation, impact reduction and early warning”. In addition, efforts and measures to achieve other and potentially conflicting SDGs will likely create synergies and/or tradeoffs for the SDG 13 targets related to adaptation. Furthermore, the SDGs and the targets need to be localized, contextualized, and adapted for successful implementation in local and regional governance agendas (GTLRG, 2016). This is also made clear by the UN. Unmasking the tradeoffs and synergies, based on broad stakeholder participation, will likely allow for a more successful implementation of the SDGs (GTLRG, 2016, Nilsson et al., 2018). However, there are major challenges associated with implementing the SDGs, including a lack of knowledge about how this should or could be done (Nilsson et al., 2018). In addition, there is limited focus on the role of participatory approaches and knowledge co-production in such processes (Glass and Newig, 2019). Given the need for participation and user involvement in localizing the SDG targets for adaptation and for resolving tradeoffs and synergies, there is a need for systematizing knowledge about how processes of co-production may contribute.

Some authors argue that the SDGs strengthen the need for societal transformation (Blythe et al., 2018, Shrivastava et al., 2020), implying a changing role for sustainability scientists from conducting problem-driven applied research to becoming agents of transformation. This is in line with Amartya Sen (2013), who argues that scientists need to engage in “informed agitation” to realize a transition to sustainability. While the co-production model has become the most widespread trope for describing joint knowledge production and for producing usable knowledge for adaptation actions, it lacks a focus on how this happens in practice, what methods for engagement of users are employed, and the extent to which such processes have an impact on policy (Dannevig et al., 2019, Mach et al., 2020). As Mach and colleagues write: “the practice of co-production is a means of changing how decisions are made by changing who is present in the knowledge-production processes” (2020: 32). In other words, processes of co-production have the potential to alter existing power relations (Eisenhauer, 2016, Scoones et al., 2020).

In the following, we categorize the reviewed literature and identify how the interplay between co-production, adaptation, and SDGs is approached. We start by clarifying the co-production concept, followed by a categorization of the research focus in the reviewed corpus. We highlight and discuss three topics based on the review: 1) the methods employed in co-production, how they localize SDG targets for adaptation and resolve synergies and tradeoffs; 2) how power asymmetries emerge through the processes of co-production; and 3) whether and how the SDG agenda contributes to a shift in the role of researchers towards a role as “informed agitators” conducting interventionist approaches for adaptation.

### 1.1 Conceptual clarifications

The main goal of sustainability sciences and adaptation research is for scientific knowledge to result in action and policy change. The failure of the so-called “linear science to policy model” to solve complex societal challenges has led to various approaches for increasing the usability of research-based knowledge through the engagement of other knowledge holders and users. Co-production has become the dominant model for such processes (Mach et al., 2020), but there are other related participatory approaches, such as “mode 2 knowledge production” (Gibbons, 1994), post-normal science (Funtowicz and Ravetz, 1994), and boundary work (Gieryn, 1983). There are several definitions, applications, and understandings of the co-production of knowledge, primarily falling into two groups. The first and most widely applied understanding of co-production of knowledge efforts is when scientists and stakeholders deliberately collaborate to produce useful knowledge. This aligns well with the abovementioned objectives and with the works of Maria Carmen Lemos and colleagues (Dilling and Lemos, 2011, Lemos, 2015). The second application is associated with Sheila Jasanoff (2004) and other scholars in the field of science technology studies (STS) and pertains to the co-production of social order that takes place when new knowledge emerges. In their review of co-production in climate research, Bremer and Meisch call the Jasanoff interpretation of co-production a descriptive lens, while the normative lens refer to approaches where co-production is deliberately facilitated to instigate action (2017). We acknowledge that the co-production of knowledge, in any shape or form, is not without challenges and pitfalls, but this discussion is beyond the scope of this paper.

Due to the conceptual ambiguity of the co-production concept, some authors have chosen to apply notions such as “joint knowledge production” (e.g., Hegger et al., 2012) or more overarching “frameworks for linking knowledge to action” (West et al., 2019).

**Fig. 1:**
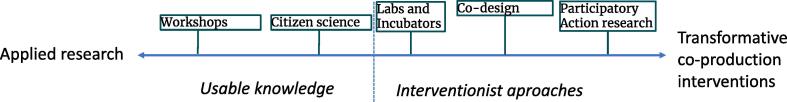
Methods of co-production found in the corpus with the least interventionist approaches to the left and the most interventionist to the right.

A substantial body of literature about researcher-stakeholder collaborations applies the terms “participatory methods” or “participatory approaches.” The purpose is to include stakeholders and knowledge users and holders in the research process, not as study objects, but as active co-producers of knowledge (Cvitanovic et al., 2019). The terms are nearly identical to the normative interpretation of co-production (e.g., Cvitanovic et al., 2019, Turnhout et al., 2020). However, there is a distinction in the literature between co-production of knowledge approaches where the intended outcome is usable knowledge, and processes of co-production that aim for system change or change in practices as part of the process (e.g., Mach et al., 2020). The latter approach would rely on interventions in the system of interest, mostly in the form of action research (e.g., Kindon et al., 2007), but also labs, policy dialogue platforms, and citizen science approaches may take the form of interventions and instigate system or policy change as part of a co-production process. The outcome of such processes is thus both usable knowledge and system change. We, therefore, designate these types of co-production approaches as “co-production interventions.” Inspired by Mach and colleagues (2020), we categorize processes of co-production for adaptation and the SDGs based on the model below—on a scale from “applied research”—using co-production to produce usable knowledge for “transformative co-production interventions” that lead to system or policy change. Fig. 1 illustrates the methods for co-production found in this review on a scale from applied research to transformative co-production interventions.

## 2 Method

We selected studies from the literature that show how processes of knowledge co-production can be conducted to increase the likelihood of adaptation actions, and those that link adaptation to the SDGs. The three objectives of the article, 1) how co-production approaches localize SDG targets and address tradeoffs and synergies, 2) whether they focus on power asymmetries and political dimensions in such participatory processes, and 3) whether and how the literature show that the SDG agenda contributes to a shift in the role of researchers, involve an evaluation of the outcome of processes of co-production for adaptation. This being a review article, the basis of this evaluation is the corpus of literature. The authors did not engage with participants in the co-production processes outlined in the corpus.

We reviewed articles in peer-reviewed scientific journals and books on the co-production of knowledge for adaptation that treated adaptation as an SDG target and/or discussed adaptation in relation to the SDGs. We did not include review articles. The selection was limited to articles published after the SDG agenda was implemented, targeting findings from post-SDG processes. This meant that the cut-off date for literature was 01.01.2016. Our selection included 52 publications. Of these, six were derived from a search by citation in the Scopus and Web of Science databases using this search string: “Adaptation,” “participation,” and “sustainability.” A search that consisted of “Adaptation,” “co-production,” and “SDG” yielded only two articles. We therefore conducted a search in Google Scholar, using the search string “SDG,” “co-production,” and “climate change adaptation.” This gave 280 hits. Of these, 44 were selected based on title, publication type (only peer-reviewed journal articles, book sections, and books were to be included), and review of abstracts. As Google Scholar search also yields results based on the occurrence of search words in the full text, an abstract review is necessary to assess whether the different documents that appear in the search are within the scope of the literature for the review. The most frequent reason for excluding documents was that they did not cover all three topics of co-production, SDGs, and adaptation. See supplementary material for the full reference list of the corpus.

The corpus was coded by two of the authors, using tables with columns for each codes. The codes were developed in an inductive manner. First a set of codes were derived from the articles’ objectives stated above. After an initial review of a subset of the corpus the redundant codes were removed, while others were added to provide more detail. The final set of codes included “field of scholarship”; “methods for co-production”; “motivation for co-production”; “SDGs addressed”; “adaptation theme”; “synergies and tradeoffs between SDGs”; “localizing efforts of SDGs”; and “power asymmetries addressed”.

## 3 Results

### 3.1 Overview of literature on climate adaptation, SDGs, and participatory approaches to knowledge co-production

**Fig. 2:**
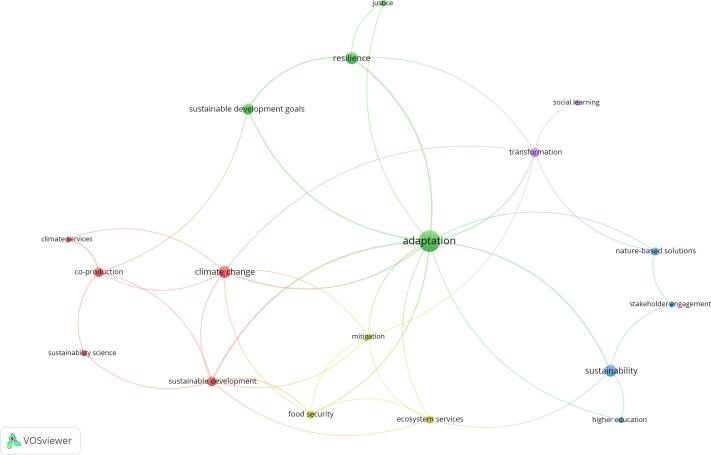
Relationships between keywords provided in the reviewed articles. Created with VOSviewer (van Eck and Waltman, 2010). The size of the circle is proportional to the occurrence of the keyword, while links represent keywords used together in at least two publications. The colors indicate the major themes in the corpus as a function of occurrences and relations of keywords in the publications.

The review identified the connections between the SDGs, adaptation to climate change and co-production of knowledge, and/or participatory approaches in multiple strands of the literature post-2016. The most frequent occurrence of this combination was found in the literature about urban resilience and planning (n = 8) and transformation (n = 5), followed by adaptation governance (n = 4) and agriculture (n = 4). This is followed by water management (n = 3), land use management (n = 3), and development studies (n = 3). The rest was distributed across related fields, with outliers in public health (n = 1) and economics (n = 1). The most frequent keywords “adaptation,” “sustainability” and “resilience” and co-occurrences of these between articles are illustrated in Fig. 2. The number and diversity of different keywords shown in Fig. 2 illustrate how mainstream both the term co-production has become and the importance of contextualizing research on adaptation within a broader context of sustainability and SDG targets.

In line with findings in other reviews, we find that the way co-production is applied and defined varies (Norström et al., 2020, Bremer and Meisch, 2017). There is no systematic alignment between variants of co-production and participatory approaches and subfields of adaptation research (adaptation in agriculture, urban planning, water management, forestry, etc.). All the reviewed articles applied co-production in a normative, prescriptive sense (Bremer and Meisch, 2017). Co-production is applied in approaches arguing for transdisciplinarity, which is particularly evident in literature that places adaptation in the context of sustainability sciences (e.g., Butler et al., 2016, Scoones et al., 2020). Furthermore, we find that co-production tends to be mentioned or applied in literature about transformation to sustainability, where adaptation is one of several core issues (Sterling et al., 2017, Scoones et al., 2020; Baum et al., 2021).

A subsection of the corpus (n = 5) deals with localization of SDG targets, resolving tradeoffs, and ensuring synergies and benefits. Four out of five of these articles provide in-depth analysis of relationships between various SDG goals and targets and adaptation (Reed et al., 2018; Glass and Newig, 2019, Orchard et al., 2019, Valencia et al., 2019), which will be outlined below. The limited literature on these fields implies that there is a knowledge gap on how to methodologically and theoretically link advancements in approaching SDGs in the co-production for adaptation literature.

Based on the review of SDGs and sustainable adaptation (see Table 1), we identified seven main methodological categories of co-production and other participatory approaches to knowledge production. These include labs, workshops (stakeholder, scenario, participatory), science-policy platforms, task groups, participatory action research, networks and incubators, and co-design of policy solutions. By far, the most widely used methods for co-production are workshops of different configurations. We argue that if this method is the sole participatory measure for co-production, it is not likely that the outcome will have a transformative impact on the system of interest. A combination of methods, however, as listed in Table 1, will more likely result in a long-lasting transformative impact. Fig. 1 shows how we rank the degree of interventions in the different methods. Methods that are intended to instigate system change as outlined in the reviewed literature include labs, co-design, and participatory action research, and we provide a few examples of this below.

**Table 1:** Methods for co-production of knowledge found in the reviewed literature.

Method	Characteristics	Number of publications in corpus	Employed by
Labs, Incubators	Testing solutions with stakeholders	7	Hjerpe et al., 2017, Kumar et al., 2020, DeLosRios-White et al., 2020, Scoones et al., 2020; Patel et al., 2020; Boeri et al., 2017, Wamsler et al., 2020
Workshops (stakeholder-, scenario-, participatory)	Events that bring researchers and stakeholders together, employing various methods for facilitating collaboration and mutual learning	15	Boeri et al., 2017, Ajibade et al., 2016, Greenhill et al., 2020, Frantzeskaki et al., 2019, Adegun and Olusoga, 2020, Paterson et al., 2020, Zougmoré et al., 2019, Capitani et al., 2019; Chae et al., 2020; Scoones et al., 2020, Ziervogel, 2019, Butler et al., 2016, Kumar et al., 2020; Baum et al., 2021; Estrella et al., 2016
Science-policy platform	Enduring meeting places for researchers and stakeholders, similarities with networks	1	Zougmoré et al., 2019
Participatory action research	Interventionist approach where researchers engage in development of new policies or solutions	1	Trott et al., 2018
Networks	Formalized collaborations between stakeholder organizations and research institutions for exchange of knowledge	3	Nagy et al., 2017, Huber-Sannwald et al., 2020, Hellin et al., 2020
Citizen science	Stakeholders and knowledge users collect data and conduct other research tasks	1	Patel et al., 2020
Co-design	Development of solutions with stakeholders that are implemented and/or tested.	4	Kumar et al., 2020, Paterson et al., 2020; Baum et al., 2021; Boeri et al., 2017

### 3.2 Does the SDG agenda promote participatory approaches and co-production of knowledge?

Participation is a prerequisite for the co-production of knowledge and an important consideration in the discourses and practices of policy development. Although there were gaps in understanding of how participation should be conceptualized in policy-making processes for adaptation under the implementation of the MDGs, significant strides were made to increase local and female participation (Collins and Ison, 2009). The transition to the SDGs compounded the differing global and local objectives to economic development, environmental sustainability, social inclusion, and for nations to commit to “good governance” (Sachs, 2012). The SDGs come with 173 targets and 232 indicators for measuring progress for the different subgoals. To adjust and adapt the indicators and targets to national, regional, and local contexts, the localizing process necessary for subnational implementation of SDGs clearly entail processes of stakeholder participation and co-production (GTLRG, 2016). Resolving tradeoffs between SDG goals represents a “critical step” in localizing the SDGs, and potential conflicts should be assessed in relation to implementation (Valencia et al., 2019). A key to mitigating tradeoffs between SDGs is to create partnerships with a diverse set of stakeholders in the localizing process (Valencia et al., 2019). This review shows that such processes are taking place in relation to adaptation. For instance, researcher-stakeholder collaborations can be an efficient measure for resolving land use challenges and adopting SDG targets tied to biodiversity, adaptation, and mitigation (Reed et al. 2019). Co-production of knowledge also serve to reconcile tradeoffs and secure consensus between stakeholders (Reed et al., 2019). While comprehensive stakeholder involvement might mitigate tradeoffs and reduce goal conflicts, such processes are also time-consuming and drain resources. A large study of SDG implementation found that efforts to resolve tradeoffs took so much time and effort that it reduced the overall performance in achieving individual SDGs (Glass and Newig, 2019). On the other hand, good performance on participatory processes was related to a high degree of SDG achievements (Glass and Newig, 2019). Orchard and colleagues (2019), drawing on interventionist case studies in the Indian Himalayas, found that building an adaptive capacity to climate change requires that synergies between SDG goals 13, 1, 2, 5, and 15 be utilized. One example combined flood prevention and forest restoration projects, which comes with several co-benefits tied to biodiversity, enhanced crop yields, and increased livelihood diversification developed in collaborative, participatory partnerships (Orchards et al., 2019). To conclude, it appears that processes for resolving synergies and tradeoffs between SDG targets tied to adaptation and other SDGs and targets do utilize participatory approaches, but these are not always researcher-stakeholder partnerships engaging in the co-production of knowledge.

### 3.3 How can co-production reveal potentials for levelling power asymmetries through adaptation?

While some of the studies reviewed show potential for social levelling of social hierarchies or socio-economic differences through adaptation efforts (e.g., Scoones et al., 2020), the review also reveals gaps in how to facilitate such levelling and “empowerment”. Few of the studies we reviewed describe what “empowerment” or “sustainability” entails in processes of adaptation, beyond describing the potentials of the co-production process. We have identified knowledge gaps in how to evaluate the results of the implementations of adaptation and whether this leads to sustainable practices and action that empower stakeholders. Although, some studies in the corpus highlight the necessity for such an effort. In a study of several adaptation processes carried out through transformation labs in water management in Mexico and India, Scoones and colleagues (2020) concluded that these activities allowed the community to move beyond internal conflicts and collectively reflect on what they wanted to conserve. Rather, the transformation labs made it possible to frame the problem as one of “maintaining the identity and meanings they attached to the Xochimilco wetland in Mexico” (Scoones et al., 2020: 211). The process “illuminated where power is held” and how it can “be mobilized to achieve more just and sustainable development pathways” (Scoones et al., 2020: 112). The authors identify three practical considerations for transformations to sustainability and SDG realization: diverse knowledge, plural pathways, and clearly defined meanings of sustainable adaptation (Scoones et al., 2020: 1).

An exception from the patterns in the corpus is that empowerment processes were only evaluated post-implementation, which suggests various modes of “leap-frogging” the SDGs. Here leap-frogging refers to social and technological advancements that bypass certain barriers to improve human and environmental outcomes and that demonstrate adaptive capabilities (Butler et al., 2016).

Co-production of adaptation solutions can also be coupled with poverty alleviation. In a study in Nusa Tenggara Barat Province, Indonesia, methodologies for engaging stakeholders in planning process in ways that addressed asymmetrical social relations and poverty was developed. Stakeholders were engaged separately and measures to manage problems tied to poverty and uneven power relations were implemented during workshops (Butler et al., 2015). The authors concluded that more sensitive methods should be established for scenario planning practices and that both an understanding and management of adaptation pathways principles should be improved (Butler et al., 2016).

Collective action is also found to increase empowerment, for instance, by lobbying for environmental issues, integrating environmental values into farming practices, increasing biodiversity, and supporting ecosystem services (Alemu et al., 2018, Orchard et al., 2019). Such cases show that encouraging such synergies can help address issues of power and politics that potentially limit adaptive governance (Karpouzoglou et al., 2016, Orchard et al., 2019).

Empowerment in marginalized communities in developing countries, fostered by strengthened social relationships and knowledge sharing, could also potentially increase the use of climate information services (CIS), which again bolster food security (McKune et al., 2018). In a project in rural communities in Senegal and Kenya, researchers made an effort to better understand access to and uptake of CIS. The project revealed that while men control decision making on food storage, women consider this issue more important than men. Women have the responsibility of using the storage more often and adapting it for new types of use, revealing how gendered discrepancies were embedded within the social structures of these agrarian communities (McKune et al., 2018). Here, empowerment refers to access to frequent and good information, but also to social connectedness with the potential for training and exchange. This example also highlights social structures where power asymmetries must be understood to facilitate usable adaptation knowledge.

Techno-scientific solutions are often applied as a response to vulnerabilities to climate change. However, as evident from studies found in this review, addressing these vulnerabilities also requires an understanding of norms, values, and socio-economic conditions (Arora, 2019, Scoones et al., 2020). In addition, such studies require a focus on power relationships between civil society actors, governments, and industry actors (Arora, 2019). This is a strong argument for a pluralistic understandings and multiple pathways towards sustainability (Arora, 2019). Although there have been many suggestions for transformation typologies, some stand out as innovative through this review. One such typology draws on “transformative engagement”*,* mostly known from the work of Stirling (2019), who, in a rejection of modernist approaches, seeks to level epistemological and cultural hierarchies. Transformative engagement rejects aspirations of control and applies more “caring practices” as key in such processes (Stirling, 2018). Caring practices signify a more holistic approach that may undo those practices that further asymmetrical power relations. Overall transformational engagement to caring practices entails what Arora (2019) argues are systems where knowledge or life is reduced or veiled, and “might enable practices to collectively become caring of vulnerable and neglected social and ecological worlds” (Arora, 2019:1575).

An example, illustrating the importance of such caring practices is the climate change adaptation efforts in the small city of Dunedin, on the south-east coast of Aotearoa New Zealand. This community is experiencing sea-level rise and increased flood events, and the potential for climate change impacts to exacerbate existing inequalities is considered high. After a flooding in 2015, a local initiative to strengthen the connection between local care networks and residents was established. The event included a biannual event and allowed for processes of care to happen during which structural relationships could be re-worked and become more just (Bond and Barth, 2020). Caring is incremental to sustainable and just adaptation, while current understandings of climate justice are too narrow in epistemological terms (Bond and Barth, 2020). Systematic critical theory, such as Marxist theory, with thick explorations of aspects such as hegemonic structures, power or hierarchy tend to overlook epistemological and cultural diversity, while the interdisciplinary framework of social-ecological systems (SES) often fails to include social systems complexity such as values and human agency. This becomes problematic in cases in where analyses of SES limits the focus and underrepresent the role of justice and power (Bond and Barth 2020).

These findings reveal important, but often veiled complexity in the strategies that address power asymmetries in co-production processes concerning SDGs. Aspects related to issues of power and climate change intersect, we argue, with epistemological (forms of knowing or understanding) and ontological (forms of being or existing) differences, where ways of knowing climate change and ways of being in the world manifest as different understandings of power, culture, science, and technology.

### 3.4 From facilitators of co-production to informed agitators—towards a new role for researchers?

While there is a broad consensus that knowledge for adaptation and other sustainability challenges needs to be co-produced, we inquire whether there also is a trend towards a more activist role for researchers, in line with Sen and others’ call for informed agitation (Sen, 2013, Shrivastava et al., 2020). The demands on sustainability researchers are significant, if we are to believe Shrivastava and colleagues (2020): “In a world beset with grand challenges, scientists must also become translators of knowledge, communicators to the public, policymakers, implementors of action, advocates of solutions, and co-designers of the future” (2020: 336). Similar calls are found in other articles, arguing that the challenges of the Anthropocene require a transformation of science itself towards more collaborative, open, and egalitarian approaches (e.g., Fazey et al., 2020). As mentioned above, there is a continuum from applied, problem-driven research where co-production efforts consist of facilitating consultation with stakeholders in a workshop – to interventionist action research where the processes of co-production also include explicit goals of changes in systems and practices (e.g Mach et al., 2020) (see Fig. 1). In the corpus reviewed for this article, 16 out of 51 articles described various types of interventionist co-production processes that either aimed at instigating system change or implemented it. Below, we outline findings from four of these: The first article outlines how intervention from a project helped foster more inclusive local governance models in South Africa, where city officials, local community stakeholders, nongovernmental organizations (NGOs), representatives, and researchers collaborated in developing local adaptation plans (Ziervogel, 2019). Another method for co-production for adaptation with an interventionist potential is through science dialogue platforms. In the second article, these have been found to be able to deliver solutions that later become institutionalized through development plans in Ghana, Senegal, and Mali for climate-smart agriculture (CSA) (Zougmore et al., 2017). Agricultural research and extension services is a field with a long history of intervention research and co-production. Networks for CSA, which established collaborations between farmers and researchers in several countries in Asia, have served to both adapt agricultural practices to climate change and to increase overall sustainability (Hellin et al., 2020). The fourth article is the only one in the corpus that presents a participatory action research approach to adaptation. It suggests a participatory action research approach for SDG 13, targeted at undergraduates in higher education (Trott et al., 2018). The article concludes that participatory action research represents a pathway for SDG realization by fostering boundary-spanning scholars that can transform higher education institutions to better serve the wellbeing of local communities (Trott et al., 2018).

Despite calls for a more activistic role by researchers, our review shows that these are not explicitly tied to—or driven by—the SDG 2030 agenda when it comes to adaptation. We second Wamsler and colleagues who calls for more interventionist research and a higher degree of science-policy co-production to forward transformative adaptation (Wamsler et al., 2017).

## 4 Knowledge gaps and directions for future research

There is an increasing emphasis on integrative participatory processes in adaptation to generate or achieve sustainable development. Evaluations of such processes should evolve in parallel with those developments. The methodological process of this review revealed many knowledge gaps in how SDGs are addressed through co-production. We have outlined three major aspects of the problematics associated with the nexus of SDGs, co-production, and adaptation. We have opened the door for further fruitful investigations and research for integrating the SDGs in other processes. We found that there is a plethora of approaches to processes of co-production of knowledge that are being carried out to further adaptation. Most of these are derived from studies of adaptation in an SDG context. We grouped them into seven distinct methods, of which different variants of workshops were the most common. Both in the literature on the sustainability science agenda in general (Sen, 2013) and the SDG agenda (Shrivastava et al., 2020), and on adaptation (e.g., Wamsler et al., 2020), there are calls for more interventionist approaches. We assessed the application of interventionist approaches in the corpus we reviewed using the scale of degrees of interventionism illustrated in Fig. 1, from “applied research” to “transformative co-production intervention.” We only found five articles presenting interventionist co-production approaches for adaptation, indicating that such approaches are not yet commonplace when doing participatory research on adaptation in the context of the SDGs or as part of SDG localizing. There are also related calls for a transformative research agenda to implement the SDGs (Fazey et al., 2020, Shrivastava et al., 2020, Wamsler et al., 2020), but the review revealed little evidence that this has taken hold in the literature. This suggests that the SDG agenda is not yet contributing to more transformative approaches in the field of climate change adaptation.

We find several examples in the literature on how tradeoffs and synergies between SDGs can be either mitigated or strengthened using participatory approaches. This includes co-production of knowledge, and several studies also show how processes of co-production have the potential to level the inequities and power asymmetries in adaptation (e.g., Sterling et al., 2017, Orchard et al., 2019, Scoones et al., 2020). Our review also revealed how caring practices can serve to level epistemological and cultural hierarchies, empowering marginalized groups for transformative adaptation. (Stirling, 2019, Arora, 2019, Bond and Barth, 2020) A striking observation is that while these articles emphasize that a co-production approach is imperative for adaptation to be transformative and sustainable, for levelling power asymmetries and empowering marginalized groups, they do not address the “social” SDGs, such as SDG 5 Gender Equality or SDG 10 Reduced Inequalities. This is clearly a missed opportunity to discuss and highlight synergies between SDGs.

The most salient aspect that links the three topics —adaptation, co-production, and the SDGs— together, are processes of adapting and localizing the adaptation and other SDG targets at the local and regional levels, while simultaneously mitigating tradeoffs and strengthening synergies. A co-production approach seems crucial for such localizing processes to be successful (e.g., Glass and Newig, 2019; Reed et al., 2019; Valencia et al., 2019).

Finally, it is important to note that, compared to the vast literature on adaptation, the co-production of knowledge, and the SDGs, our review found that very few of these studies address these in combination. Naturally, this limits the generalizability and robustness of our findings, but more importantly, it reflects a significant gap in research on the tradeoffs and synergies between the three critical aspects of societal sustainability and transformation.

## Declaration of Competing Interest

The authors declare that they have no known competing financial interests or personal relationships that could have appeared to influence the work reported in this paper.
