# Supplementary material for: Advancements of sustainable development goals in co-production for climate change adaptation research
Source: Clim Risk Manag. 2022;36:None. doi: 10.1016/j.crm.2022.100438 (PMC9164297; doi:10.1016/j.crm.2022.100438)
Supplement: Supplementary Data 1 [file mmc1.docx]

Supplementary material

List of literature reviewed (the corpus)

Ajibade, I., Pelling, M., Agboola, J., Garschagen, M., 2017. Sustainability Transitions: Exploring Risk Management and the Future of Adaptation in the Megacity of Lagos. https://doi.org/10.1142/S2345737616500093 03, 1650009. https://doi.org/10.1142/S2345737616500093

Anderson, V., Gough, W.A., Agic, B., 2021. Nature-based equity: An assessment of the public health impacts of green infrastructure in Ontario Canada. Int. J. Environ. Res. Public Health 18. https://doi.org/10.3390/ijerph18115763

Baum, D., Yagüe-Blanco, J.L., Escobar, J., 2021. Capacity development strategy empowering the decentralized governments of Ecuador towards local climate action. J. Clean. Prod. 285. https://doi.org/10.1016/j.jclepro.2020.125320

Berbés-Blázquez, M., Mitchell, C.L., Burch, S.L., Wandel, J., 2017. Understanding climate change and resilience: assessing strengths and opportunities for adaptation in the Global South. Clim. Change 141, 227–241. https://doi.org/10.1007/s10584-017-1897-0

Boeri, A., Longo, D., Gianfrate, V., Lorenzo, V., 2017. Resilient communities. Social infrastructures for sustainable growth of urban areas. A case study. Int. J. Sustain. Dev. Plan. 12, 227–237. <https://doi.org/10.2495/SDP-V12-N2-227-237>

Bond, S., Barth, J. 2020. Care-full and just: Making a difference through climate change adaptation. Cities, 102, 102734.

Borie, M., Pelling, M., Ziervogel, G., Hyams, K., 2019. Mapping narratives of urban resilience in the global south. Glob. Environ. Chang. 54, 203–213. https://doi.org/10.1016/j.gloenvcha.2019.01.001

Butler, J.R.A., Bohensky, E.L., Suadnya, W., Yanuartati, Y., Handayani, T., Habibi, P., Puspadi, K., Skewes, T.D., Wise, R.M., Suharto, I., Park, S.E., Sutaryono, Y., 2016. Scenario planning to leap-frog the Sustainable Development Goals: An adaptation pathways approach. Clim. Risk Manag. 12, 83–99. https://doi.org/10.1016/j.crm.2015.11.003

Byskov, M.F., Hyams, K., Satyal, P., Anguelovski, I., Benjamin, L., Blackburn, S., Borie, M., Caney, S., Chu, E., Edwards, G., Fourie, K., Fraser, A., Heyward, C., Jeans, H., McQuistan, C., Paavola, J., Page, E., Pelling, M., Priest, S., Swiderska, K., Tarazona, M., Thornton, T., Twigg, J., Venn, A., 2019. An agenda for ethics and justice in adaptation to climate change. Clim. Dev. https://doi.org/10.1080/17565529.2019.1700774

Capitani, C., Garedew, W., Mitiku, A., Berecha, G., Hailu, B.T.B.T., Heiskanen, J., Hurskainen, P., Platts, P.J.P.J., Siljander, M., Pinard, F., Johansson, T., Marchant, R., 2019. Views from two mountains: exploring climate change impacts on traditional farming communities of Eastern Africa highlands through participatory scenarios. Sustain. Sci. 14, 191–203. https://doi.org/10.1007/s11625-018-0622-x

Castella, J.-C., Lestrelin, G., 2021. Exploring the environmental impact of agrarian changes in Southeast Asia through participatory evaluation of ecosystem services | Explorer l’impact environnemental des transformations agraires en Asie du Sud-Est grâce à l’évaluation participative des serv. Cah. Agric. 30. https://doi.org/10.1051/cagri/2020042

Chae, Y., Choi, S.H., Kim, Y.J., 2020. Climate change policy implications of sustainable development pathways in Korea at sub-national scale. Sustain. 12. https://doi.org/10.3390/su12104310

Cremades, R., Mitter, H., Tudose, N.C., Sanchez-Plaza, A., Graves, A., Broekman, A., Bender, S., Giupponi, C., Koundouri, P., Bahri, M., Cheval, S., Cortekar, J., Moreno, Y., Melo, O., Karner, K., Ungurean, C., Davidescu, S.O., Kropf, B., Brouwer, F., Marin, M., 2019. Ten principles to integrate the water-energy-land nexus with climate services for co-producing local and regional integrated assessments. Sci. Total Environ. 693. https://doi.org/10.1016/j.scitotenv.2019.133662

Daniels, E., Bharwani, S., Gerger Swartling, Å., Vulturius, G., Brandon, K., 2020. Refocusing the climate services lens: Introducing a framework for co-designing “transdisciplinary knowledge integration processes” to build climate resilience. Clim. Serv. 19, 100181. https://doi.org/10.1016/j.cliser.2020.100181

David Tàbara, J., Jäger, J., Mangalagiu, D., Grasso, M., 2018. Defining transformative climate science to address high-end climate change. Reg. Environ. Chang. 2018 193 19, 807–818. https://doi.org/10.1007/S10113-018-1288-8

DeLosRios-White, M., Roebeling, P., Valente, S, 2020. Mapping the Life Cycle Co-Creation Process of Change Adaptation. Resources 34.

Donkor, F.K., 2017. Social Learning as a Vehicle for Complementary Strategies in Forest Resource Management. J. Asian Res. 1, 71. https://doi.org/10.22158/jar.v1n1p71

Eisenhauer, D.C., 2016. Pathways to Climate Change Adaptation: Making Climate Change Action Political. Geogr. Compass 10, 207–221. https://doi.org/10.1111/gec3.12263

Estrella, M., Renaud, F.G., Sudmeier-Rieux, K., Nehren, U., 2016. Defining New Pathways for Ecosystem-Based Disaster Risk Reduction and Adaptation in the Post-2015 Sustainable Development Agenda. Adv. Nat. Technol. Hazards Res. 42, 553–591. https://doi.org/10.1007/978-3-319-43633-3_24

Frantzeskaki, N., Hölscher, K., Holman, I.P., Pedde, S., Jaeger, J., Kok, K., Harrison, P.A., 2019. Transition pathways to sustainability in greater than 2 °C climate futures of Europe. Reg. Environ. Chang. 19, 777–789. https://doi.org/10.1007/s10113-019-01475-x

Ghosh, A., 2018. For the ‘Comfortably Numb: Conclusion. Adv. Asian Human-Environmental Res. 217–237. https://doi.org/10.1007/978-3-319-63892-8_7

Glass, L.-M., Newig, J., 2019. Governance for achieving the Sustainable Development Goals: How important are participation, policy coherence, reflexivity, adaptation and democratic institutions? Earth Syst. Gov. 2, 100031. https://doi.org/10.1016/J.ESG.2019.100031

Greenhill, L., Kenter, J.O., Dannevig, H., 2020. Adaptation to climate change–related ocean acidification: An adaptive governance approach. Ocean Coast. Manag. 191, 105176. https://doi.org/10.1016/j.ocecoaman.2020.105176

Hart, D.D., Bell, K.P., Lindenfeld, L.A., Jain, S., Johnson, T.R., Ranco, D., McGill, B., 2015. Strengthening the role of universities in addressing sustainability challenges: The Mitchell Center For Sustainability Solutions as an institutional experiment. Ecol. Soc. 20. https://doi.org/10.5751/ES-07283-200204

Hellin, J., Balié, J., Fisher, E., Kohli, A., Connor, M., Yadav, S., Kumar, V., Krupnik, T.J., Sander, B.O., Cobb, J., Nelson, K., Setiyono, T., Puskur, R., Chivenge, P., Gummert, M., 2020. Trans-Disciplinary Responses to Climate Change: Lessons from Rice-Based Systems in Asia. Climate 8, 35. https://doi.org/10.3390/cli8020035

Huber-Sannwald, E., Martinez-Tagüeña, N., Espejel, I., Lucatello, S., Coppock, D.L., Gómez, V.M.R., 2020. Introduction: International Network for the Sustainability of Drylands—Transdisciplinary and Participatory Research for Dryland Stewardship and Sustainable Development, in: Stewardship of Future Drylands and Climate Change in the Global South. Springer, pp. 1–24.

Karki, M., 2017. Need for Transformative Adaptation in South Asia. Int. J. Multidiscip. Stud. 4, 1. https://doi.org/10.4038/ijms.v4i2.17

King, E.G., Nelson, D.R., McGreevy, J.R., 2019. Advancing the integration of ecosystem services and livelihood adaptation. Environ. Res. Lett. 14. https://doi.org/10.1088/1748-9326/ab5519

Kumar, P., Johnson, B.A., Dasgupta, R., Avtar, R., Chakraborty, S., Kawai, M., Magcale-Macandog, D.B., 2020. Participatory approach for more robust water resource management: Case study of the santa rosa sub-watershed of the Philippines. Water (Switzerland) 12. https://doi.org/10.3390/W12041172

Kumar, P., Debele, S.E., Sahani, J., Aragão, L., Barisani, F., Basu, B., Bucchignani, E., Charizopoulos, N., Di Sabatino, S., Domeneghetti, A., Edo, A.S., Finér, L., Gallotti, G., Juch, S., Leo, L.S., Loupis, M., Mickovski, S.B., Panga, D., Pavlova, I., Pilla, F., Prats, A.L., Renaud, F.G., Rutzinger, M., Basu, A.S., Shah, M.A.R., Soini, K., Stefanopoulou, M., Toth, E., Ukonmaanaho, L., Vranic, S., Zieher, T., 2020. Towards an operationalisation of nature-based solutions for natural hazards. Sci. Total Environ. 731, 138855. https://doi.org/10.1016/j.scitotenv.2020.138855

McElwee, P., Calvin, K., Campbell, D., Cherubini, F., Grassi, G., Korotkov, V., Hoang, A. Le, Lwasa, S., Nkem, J., Nkonya, E., Saigusa, N., Soussana, J.-F., Taboada, M.A., Manning, F., Nampanzira, D., Smith, P., 2020. The impact of interventions in the global land and agri-food sectors on Nature’s Contributions to People and the UN Sustainable Development Goals. Glob. Chang. Biol. 26, 4691–4721. https://doi.org/10.1111/GCB.15219

McKune, S., Poulsen, L., Russo, S., Devereux, T., Faas, S., McOmber, C., Ryley, T., 2018. Reaching the end goal: Do interventions to improve climate information services lead to greater food security? Clim. Risk Manag. 22, 22–41. https://doi.org/10.1016/j.crm.2018.08.002

Nagy, G.J., Cabrera, C., Coronel, G., Aparicio-Effen, M., Arana, I., Lairet, R., Villamizar, A., 2017. Addressing climate adaptation in education, research and practice: the CLiVIA-network. Int. J. Clim. Chang. Strateg. Manag. 9, 469–487. https://doi.org/10.1108/IJCCSM-04-2016-0056

Nocito, E.S., Brooks, C.M., Strong, A.L., 2020. Gazing at the Crystal Ball: Predicting the Future of Marine Protected Areas Through Voluntary Commitments. Front. Mar. Sci. 6, 1–13. https://doi.org/10.3389/fmars.2019.00835

Orchard, S., Glover, D., Thapa Karki, S., Ayele, S., Sen, D., Rathod, R., Rowhani, P., 2019. Exploring synergies and trade-offs among the sustainable development goals: collective action and adaptive capacity in marginal mountainous areas of India. Sustain. Sci. https://doi.org/10.1007/s11625-019-00768-8

Patel, Z., Marrengane, N., Smit, W., Anderson, P.M.L., 2020. Knowledge Co-production in Sub-Saharan African Cities: Building Capacity for the Urban Age 189–214. https://doi.org/10.1007/978-981-15-5358-5_8

Paterson, S.K., Le Tissier, M., Whyte, H., Robinson, L.B., Thielking, K., Ingram, M., McCord, J., 2020. Examining the Potential of Art-Science Collaborations in the Anthropocene: A Case Study of Catching a Wave. Front. Mar. Sci. 7, 1–13. https://doi.org/10.3389/fmars.2020.00340

Pundt, H., Heilmann, A., 2020. Building Collaborative Partnerships: An Example of a 3rd Mission Activity in the Field of Local Climate Change Adaptation. World Sustain. Ser. 621–636. https://doi.org/10.1007/978-3-030-15604-6_38

Reed, J., Barlow, J., Carmenta, R., van Vianen, J., Sunderland, T., 2019. Engaging multiple stakeholders to reconcile climate, conservation and development objectives in tropical landscapes. Biol. Conserv. 238, 108229. https://doi.org/10.1016/J.BIOCON.2019.108229

Sanchez Rodriguez, R., Ürge-Vorsatz, D., Barau, A.S., 2018. Sustainable Development Goals and climate change adaptation in cities. Nat. Clim. Chang. 2018 83 8, 181–183. https://doi.org/10.1038/s41558-018-0098-9

Scoones, I., Stirling, A., Abrol, D., Atela, J., Charli-Joseph, L., Eakin, H., Ely, A., Olsson, P., Pereira, L., Priya, R., van Zwanenberg, P., Yang, L., 2020. Transformations to sustainability: combining structural, systemic and enabling approaches. Curr. Opin. Environ. Sustain. https://doi.org/10.1016/J.COSUST.2019.12.004

Sterling, E.J., Filardi, C., Toomey, A., Sigouin, A., Betley, E., Gazit, N., Newell, J., Albert, S., Alvira, D., Bergamini, N., Blair, M., Boseto, D., Burrows, K., Bynum, N., Caillon, S., Caselle, J.E., Claudet, J., Cullman, G., Dacks, R., Eyzaguirre, P.B., Gray, S., Herrera, J., Kenilorea, P., Kinney, K., Kurashima, N., Macey, S., Malone, C., Mauli, S., McCarter, J., McMillen, H., Pascua, P., Pikacha, P., Porzecanski, A.L., de Robert, P., Salpeteur, M., Sirikolo, M., Stege, M.H., Stege, K., Ticktin, T., Vave, R., Wali, A., West, P., Winter, K.B., Jupiter, S.D., 2017. Biocultural approaches to well-being and sustainability indicators across scales. Nat. Ecol. Evol. 2017 112 1, 1798–1806. https://doi.org/10.1038/s41559-017-0349-6

Trott, C.D., Weinberg, A.E., McMeeking, L.B.S., 2018. Prefiguring sustainability through participatory action research experiences for undergraduates: Reflections and recommendations for student development. Sustain. 10, 1–21. https://doi.org/10.3390/su10093332

Valencia, S.C., Simon, D., Croese, S., Nordqvist, J., Oloko, M., Sharma, T., Taylor Buck, N., Versace, I., 2019. Adapting the Sustainable Development Goals and the New Urban Agenda to the city level: Initial reflections from a comparative research project. Int. J. Urban Sustain. Dev. 11, 4–23. https://doi.org/10.1080/19463138.2019.1573172

Wamsler, C., Wickenberg, B., Hanson, H., Alkan Olsson, J., Stålhammar, S., Björn, H., Falck, H., Gerell, D., Oskarsson, T., Simonsson, E., Torffvit, F., Zelmerlow, F., 2020. Environmental and climate policy integration: Targeted strategies for overcoming barriers to nature-based solutions and climate change adaptation. J. Clean. Prod. 247. https://doi.org/10.1016/j.jclepro.2019.119154

Ward, S., Staddon, C., De Vito, L., Zuniga-Teran, A., Gerlak, A.K., Schoeman, Y., Hart, A., Booth, G., 2019. Embedding social inclusiveness and appropriateness in engineering assessment of green infrastructure to enhance urban resilience. Urban Water J. 16, 56–67. https://doi.org/10.1080/1573062X.2019.1633674

West, S., van Kerkhoff, L., Wagenaar, H., 2019. Beyond linking knowledge and action: towards a practice-based approach to transdisciplinary sustainability interventions. POLICY Stud. 40, 534–555. https://doi.org/10.1080/01442872.2019.1618810

White, D.D., Lawless, K.L., Vivoni, E.R., Mascaro, G., Pahle, R., Kumar, I., Coli, P., Castillo, R.M., Moreda, F., Asfora, M., 2019. Co‐Producing Interdisciplinary Knowledge and Action for Sustainable Water Governance: Lessons from the Development of a Water Resources Decision Support System in Pernambuco, Brazil. Glob. Challenges 3, 1800012. https://doi.org/10.1002/gch2.201800012

Ziervogel, G., 2019. Building transformative capacity for adaptation planning and implementation that works for the urban poor: Insights from South Africa. Ambio 48, 494–506. https://doi.org/10.1007/s13280-018-1141-9

Zougmoré, R.B., Partey, S.T., Totin, E., Ouédraogo, M., Thornton, P., Karbo, N., Sogoba, B., Dieye, B., Campbell, B.M., 2019. Science-policy interfaces for sustainable climate-smart agriculture uptake: lessons learnt from national science-policy dialogue platforms in West Africa. Int. J. Agric. Sustain. 17, 367–382. https://doi.org/10.1080/14735903.2019.1670934
